# Enhancing abdominal wall healing using an oriented polycaprolactone microfibrous scaffold prepared using the fiber drawing method: A rabbit model study

**DOI:** 10.1007/s10029-025-03544-z

**Published:** 2026-01-16

**Authors:** Michala Klusáček Rampichová, Kateřina Strnadová, M. Plencner, L. Stanislav, A. Litvinec, Z. Tonar, T. Blassová, M. Otáhal, E. Filová, D. Lukáš, V. Jenčová

**Affiliations:** 1 Department of Tissue Engineering, Institute of Experimental Medicine, The Czech Academy of Sciences, Prague, Czech Republic; 2 Department of Chemistry, Faculty of Sciences, Humanities and Education, Technical University of Liberec, Liberec, Czech Republic; 3 Faculty of Mechanical Engineering, Technical University of Liberec, Liberec, Czech Republic; 4 Department of Histology and Embryology and Biomedical Centre, Faculty of Medicine in Pilsen, Charles University, Pilsen, Czech Republic; 5 Department of Natural Sciences, Faculty of Biomedical Engineering, Czech Technical University, Kladno, Czech Republic; 6 Institute of Experimental Medicine, Prague 4, Videnska, 1083, 14220 Czech Republic; 7 Technical University of Liberec, Studentská 1402/2, 461 17 Liberec, Czech Republic

**Keywords:** Tissue scaffolds, Manufacturing processes, Surgical wound healing, Abdominal wall, Rabbits, Fiber drawing method

## Abstract

**Purpose:**

Incisional hernia is a common postoperative complication following abdominal surgery. Despite the use of synthetic meshes, recurrence rates remain high. This study aimed to develop and evaluate a biodegradable, aligned microfibrous scaffold to support wound healing and strengthen abdominal wall repair.

**Methods:**

Scaffolds were fabricated from poly(ε-caprolactone) (PCL) using a controlled fiber-drawing technique to produce highly aligned microfibers with reproducible thickness and architecture. Their biocompatibility was examined in vitro using fibroblasts through adhesion and proliferation assays. For in vivo evaluation, the scaffolds were implanted over standardized abdominal wall incisions in rabbits. After six weeks, the regenerated tissue was harvested for mechanical testing to determine tensile strength and elasticity, while histological and immunohistochemical analyses assessed collagen type I deposition and neovascularization within the scaffold area.

**Results:**

The aligned PCL scaffold promoted strong cell attachment and proliferation in vitro. In vivo, its application significantly increased tensile modulus compared with control wounds. Histological analysis revealed denser and more organized collagen deposition and a higher microvessel density in the scaffold-treated group, indicating enhanced tissue remodeling and vascular integration.

**Conclusion:**

The aligned PCL microfibrous scaffold improved the mechanical and biological quality of the abdominal wall healing in vivo. These results suggest its potential for reducing the formation of incisional hernias and are suitable for further testing leading to use in clinical practice.

**Graphical Abstract:**

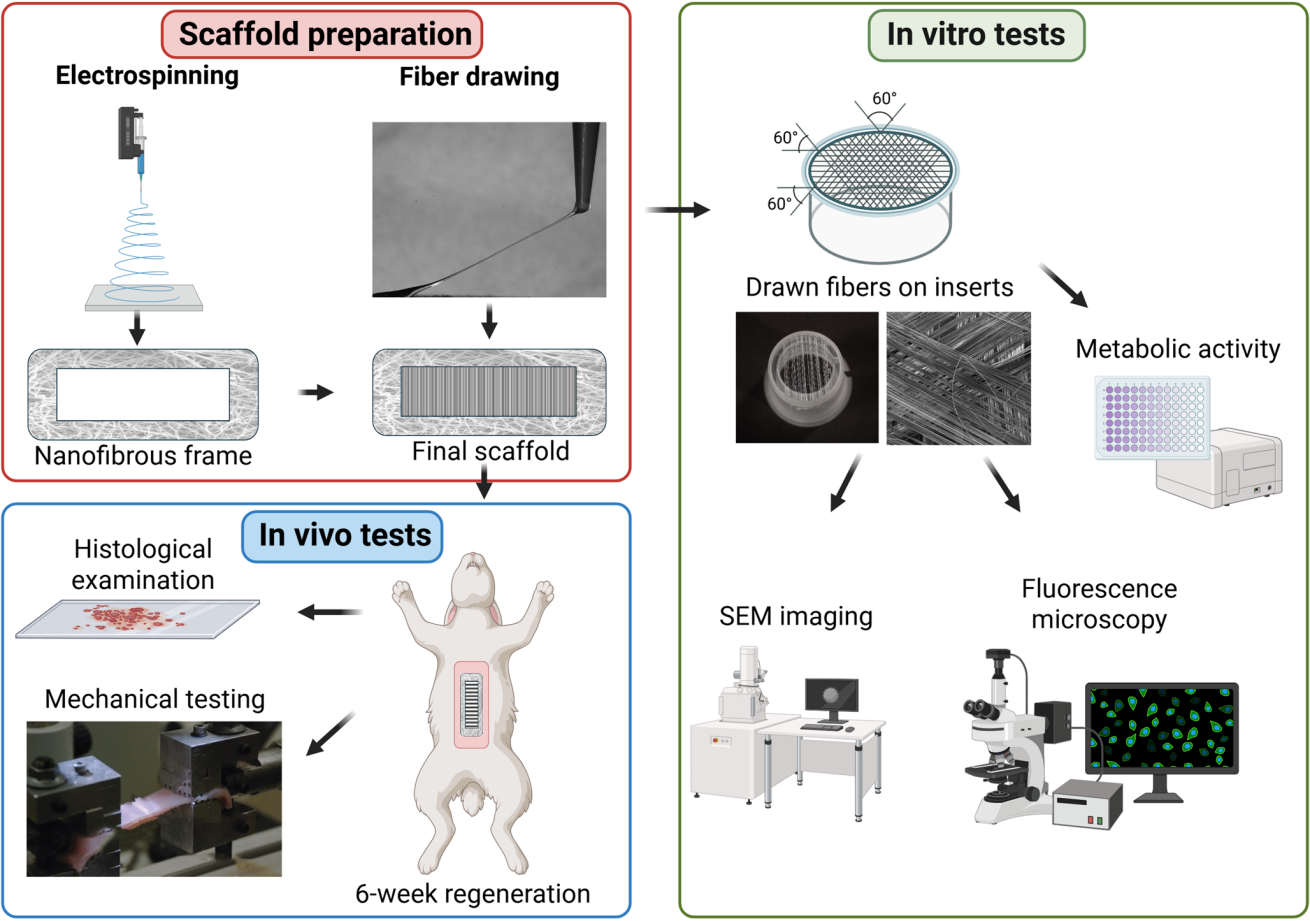

**Supplementary Information:**

The online version contains supplementary material available at 10.1007/s10029-025-03544-z.

## Introduction

Incisional hernia is a common postoperative complication. It occurs after abdominal surgery, as a result of incomplete healing of the incision or thinning of the abdominal wall in close proximity to the incision. It develops in 2–20% of patients [1]. Treatment of a developed hernia consists of repairing the abdominal wall and its reinforcement with hernia mesh [2]. A variety of surgical meshes from absorbable and non-absorbable materials are commercially available [3]. However, even after corrective surgery, recurrence occurs in 8.7–32% of patients. This variance is due to factors such as patient characteristics, the use of meshes and the surgical technique [4]. A high recurrence rate of between 25 and 32% is observed even when using surgical meshes after 5 to 10 years [5]. For this reason, new strategies are being sought in the treatment and prevention of incisional hernia. In addition to refining repair techniques, it is advisable to focus on prevention by promoting proper incision healing in at-risk patients. The most reported risk factors for hernia include obesity, abdominal aneurysm, gender, and age [6, 7]. Other risk factors could include systemic chronic diseases such as diabetes mellitus, renal failure, smoking, and malnutrition conditions, coronary heart disease and systemic long-term medication with steroids and immunosuppressants [8–11]. In these risk groups of patients, it would be beneficial to use a material that would promote incision healing and increase the mechanical strength of the incision.

Presently, most clinical hernia repairs continue to rely on permanent synthetic meshes,—predominantly polypropylene,—due to their reliable, long-term mechanical strength and extensive clinical use However, the permanent presence of synthetic polymers can be associated with chronic foreign-body reaction, mesh contraction, adhesion formation and infection in some patients, which has driven interest in alternatives. Emerging options under active investigation include slowly absorbable “biosynthetic” polymers (e.g. P4HB, PLA/PGA blends) that provide temporary mechanical support and then resorb while the host tissue remodels, biologic extracellular-matrix scaffolds intended to integrate and be remodeled by host tissue, and hybrid/composite constructs or surface coatings that combine a synthetic backbone with bioactive or anti-adhesive modifications to reduce adverse reactions [12].

Currently, nanofibers are a popular material in tissue engineering and wound healing. This is mainly due to their morphology and fiber diameter mimicking the extracellular matrix, which supports cell adhesion and proliferation. Moreover, they are highly permeable and have a high absorption rate of the exudate formed on the wound surface. Another advantage is that nanofibers can be enriched by antibacterial drugs or stimulating agents. Many natural and synthetic polymers have been successfully used in the form of nanofibers in wound healing applications [13, 14].

Nanofibers have been used for the enhancement of surgical mesh properties [15] or as the mesh itself, with the possibility of functionalization with antibacterial molecules [16–18]. A variety of polymers, such as polycaprolactone (PCL) [18, 19], polyvinyl alcohol (PVA) [16] or PCL methacrylated fibers (PCLMA) [17] have been used as a material for nanofibrous hernia mesh. However, the use of nanofibers as hernia mesh is accompanied by limitations, such as low mechanical strength and the risk of adhesions with internal organs.

In our previous study [15], we showed that polycaprolactone (PCL) nanofibers prepared using electrospinning can serve as a tool for the enhancement of commercial polypropylene mesh integrity in regenerated tissue but can also be used as support for incision healing itself, or in combination with growth factors [15] or platelets [19]. PCL nanofibers containing growth factors enhanced the formation of collagen at the site of incision and improved its mechanical properties.

Drawing is one of the many non-woven techniques that can be used for scaffold preparation. It is a method of pulling a single fiber from a droplet of polymer solution or melt without using an electrical field [20–22] and it is possible to do it even by hand. The fibers can be made from various types of polymers, varying in diameter from nano- to micro-scale. In addition, different structures can be obtained depending on the direction of the fiber-pulling, the combination of nano- and microfibers, and the combination of polymers. With additional processing we can also obtain yarns. This is enabled due to the manipulation of a single fiber. Every single emerging fiber can be manipulated while drawing. This way we can prepare more complicated structures and patterns as has been demonstrated quite recently by R. S. Keynton and co-workers [23]. Drawing is not a common method when considering tissue engineering. One of the reasons is the low productivity of the fibers compared to other nonwoven technologies, e.g. electrospinning, forcespinning, melt-blown and others, which are often used for the fabrication of micro- or nanofibers from various types of polymers [24]. They are reasonably productive, however they can only produce the oriented structures in just one direction in one layer without allowing the possibility to manipulate the fibers, and are thus unable to provide more sophisticated fibrous structures, and, in addition, the orderliness is often not so accurate [25]. On the other hand, for these experiments we used a lab-scale machine with just one drawing needle and as such, this machine could be scaled up to multiple drawing needles with higher drawing speeds.

It is known that fiber drawing is influenced by various extrinsic parameters such as humidity, temperature, solvent evaporation, trajectory and the speed of drawing [26–28]. Also, the concentration of the polymer solution as well as the molecular weight of the polymer play a very important role in drawing [29]. These are nonetheless features which influence other fiber spinning techniques as well and when keeping the same process production conditions, the method is reasonably reproducible.

The mechanical drawing of fibers has been a known technique for many years. Even Vacanti in 1988 used drawn fibers as one of the scaffolds in his experiments [30]. Nevertheless, nowadays drawing is neither studied, nor used in tissue engineering to a great extent. The aforementioned features make drawing a suitable method for the fabrication of scaffolds for specific tissues with oriented extracellular matrix and cells. Neural tissue, muscles and tendons are good examples of such tissue. Precise orientation makesthe method also suitable for fabrication of hernia meshes.

The current study is based on the assumption that oriented drawn fibers can target the growth of the adhered cells synthesizing proteins of the extracellular matrix, and thus mechanically promote abdominal wall healing. We developed a scaffold with a specific design, where the fibers are oriented perpendicularly to the incision to stimulate cell growth and tissue formation and accelerate scar healing. PCL was chosen as a biocompatible and biodegradable polymer verified in many scientific studies and approved by the FDA for use in human medicine.

## Methods

### Preparation of scaffolds using the drawing technique

**Fig. 1:**
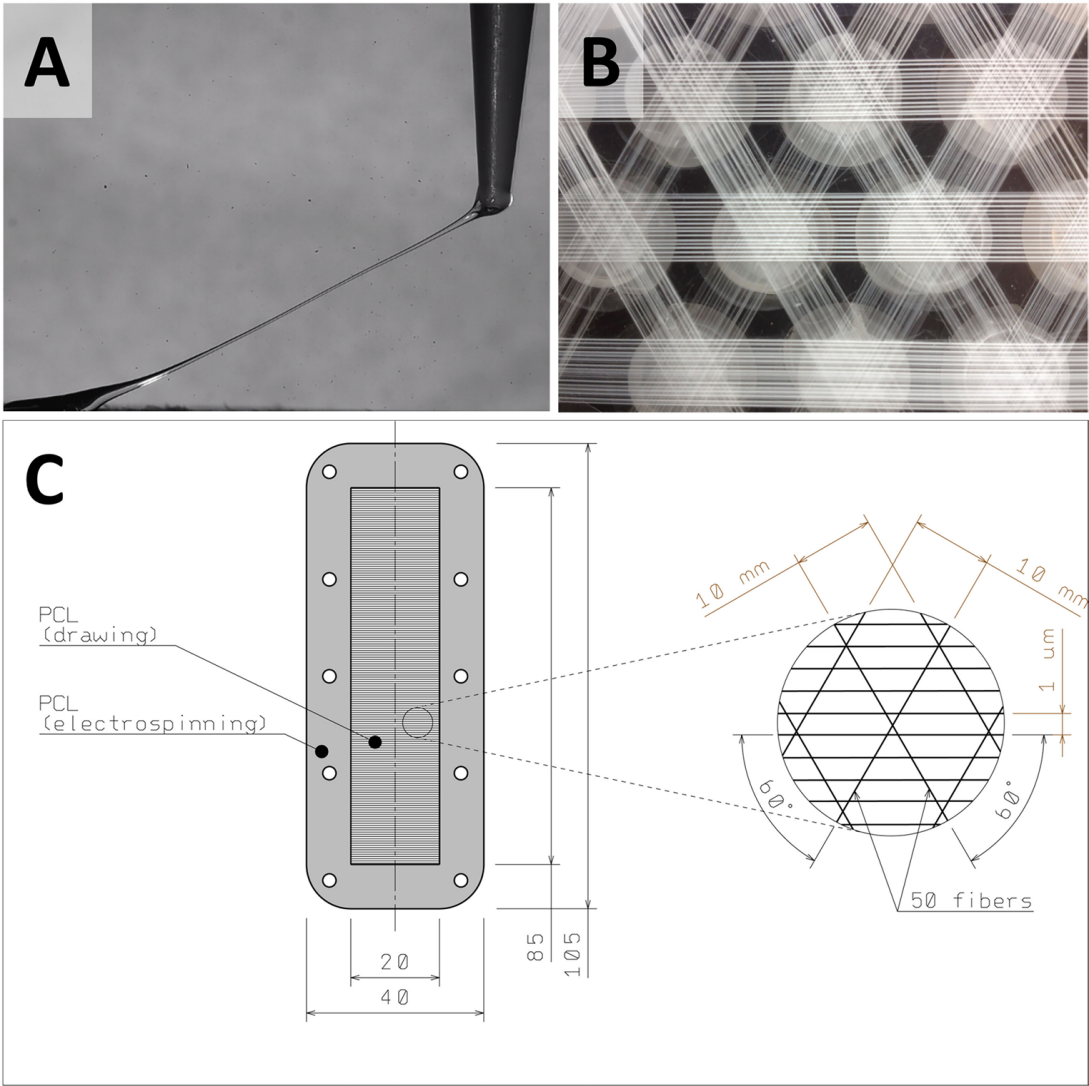
Preparation of the scaffolds for in vitro and in vivo testing. The principle of the drawing method lies in pulling a single fiber from a droplet of polymer solution (**A**). For in vitro testing, fibers were fixed on inserts fitting in wells of the 24-well plate in three directions (**B**). The scaffold for in vivo testing consisted of a nanofibrous frame on which drawn fibers were fixed in three dimensions (**C**)

The scaffolds were prepared using the mechanical drawing technique. Microfibers were prepared from poly-ε-caprolactone (PCL) with molecular weight MW 80,000 (Sigma-Aldrich, St. Louis, MO, USA). Drawing was performed from a 24 wt% solution of PCL dissolved in chloroform: ethanol solution 3:2. The motion program of the micromanipulator developed in our laboratory consisted of parabolic interpolation with an S – curve acceleration (500 ms) and deceleration (1500 ms) profile. The top speed of the drawing element was 0.08 m.s^− 1^ with 100 fibers/mm. For the first step, a droplet of a PCL solution is placed on an underlay. A movable element, which was a needle, touched the droplet and eroded its surface. After the needle was taken away the polymer started to form a fiber due to the surface tension (Fig. 1A). The PCL solution gradually solidified due to the evaporation of the solvent. Extension of the fiber is achieved by the presence of the still liquid core material, and simultaneously by the stretching of the surface. The process ended when the needle laid the fiber down on the underlay at a defined distance.

#### Preparation of drawn fibers for in vitro testing

The in vitro tests of PCL fiber biocompatibility required fixation of the fibers to the holder. For this purpose, special inserts were prepared from Poly(methyl methacrylate) (TITAN – Multiplast spol. s.r.o., Smrzovka, Czech Republic) at the Department of manufacturing systems and automation at the Technical University of Liberec. Microfibers were drawn directly onto the inserts and fixed to the edge of the ring oriented in three different directions with a mutual angle of 60° (Fig. 1B). The fiber density was set at 1,000 fibers per centimeter for each layer. The size of the inserts was designed to fit in a 24-well plate and allowed better manipulation with the fibers as well as keeping the scaffolds in the designed pattern (Fig. 1B). Immobilized microfibers were sterilized in 70% ethanol for 30 min and washed several times in sterile phosphate buffer saline (PBS, pH 7.4) prior to cell seeding.

#### Preparation of the scaffold for in vivo testing

PCL microfibers were manufactured using the drawing technique as described previously. For the in vivo samples, the drawn fibers were fixed to a specific frame prepared by the electrospinning from the 16 wt% solution of PCL with molecular weight MW 45,000 (Sigma-Aldrich, St. Louis, MO, USA), dissolved in chloroform: ethanol with a ratio of 9:1. A high-voltage source generated voltages of up to 45 kV, and the polymer solution was connected to the high-voltage source. Electrospun nanofibers were deposited on the grounded collecting electrode. The rectangular frame had an internal dimension of 85 × 20 mm and an external dimension of 105 × 40 mm. The frame was reinforced with drops of PCL polymer solution (5 drops at each side of the frame) to prevent tearing of the nanofibrous frame during its fixation to the abdominal wall with asurgical suture. The scaffold fibers were laid perpendicular to the long axis of the nanofiber grid with a spacing of 1 μm. The resulting density was therefore 1000 fibers per cm. The fiber layer was reinforced with bundles of 50 fibers running at an angle of 60° to the long axis of the scaffold. (Fig. 1C). The scaffolds were sterilized using ethylene oxide at 37 °C and were used one week after sterilization, to air out possible remnants of ethylene oxide.

#### Characterization of the scaffolds

The drawn microfibers were visualized using scanning electron microscopy (SEM). The samples were sputter-coated with a layer of gold approximately 5 nm in thickness using a Rotary-Pumped Sputter Coater (Q150R, Quorum Technologies Ltd, East Grinstead, UK). The samples were examined using a VEGA3 SB – EasyProbe electron microscope (Tescan, Brno, Czech Republic). The fiber diameters were measured from an arbitrarily selected section of SEM images using NIS Elements software (Nikon Instruments Europe B.V., Amsterdam, Netherlands). The distribution of the fiber diameters was determined quantitatively from 200 measurements.

### In vitro test of biocompatibility

#### Cell seeding and culture

The fibers fixed on inserts were tested using mouse 3T3 fibroblasts (line 3T3-Swiss albino CCL-92™, ATCC, Manassas, VA, USA), cultured in Dubelco’s Modified Eagle’s Medium (DMEM; Lonza, Basel, Switzerland) supplemented with 10% (v/v) fetal bovine serum (FBS; PAA Laboratories GmbH, Pasching, Austria) and 1% (v/v) penicillin/streptomycin/amphotericin B (Lonza, Basel, Switzerland). The cells were seeded on the fibers at a density of 1 × 10^5^ per well of a 24-well plate. 24 h after seeding, the inserts with fibers were transferred to the new wells to eliminate any contribution from non-adherent cells. The medium during the in vitro testing was changed every third day.

#### Evaluation of cell metabolic activity

Cell metabolic activity was measured using the MTT assay on days 3, 7, and 21, respectively. 50 µl of MTT (3-[4,5-dimethylthiazol-2-yl]−2,5-diphenyltetrazolium bromide, 1 mg/mL; Sigma-Aldrich, St. Louis, MO, USA) in PBS (pH 7.4) was added to 150 µL of the fresh medium and incubated with samples for 3 h. Using mitochondrial dehydrogenase of normally metabolizing cells, the MTT was reduced to purple formazan. The formazan crystals were solubilized with 200 µL of acidic isopropyl alcohol (IPA, pH 1; Penta spol. s.r.o., Prague, Czech Republic). The absorbance was measured on a microplate reader (ELx808 Absorbance reader; BioTek, Winooski VT, USA) at 570 nm and reference wavelength 650 nm.

#### Cell distribution and proliferation analysis

Cell distribution and proliferation was evaluated using SEM and fluorescent microscopy with DAPI (4’,6-diamidino-2-phenylindole; Sigma-Aldrich, St. Louis, MO, USA) staining on days 7, 14, and 21. The scaffolds were washed with PBS to remove non-adherent cells and fixed with 2,5% glutaraldehyde (Sigma-Aldrich, St. Louis, MO, USA). After the fixation the samples for SEM were dried up with an upgrading concentration of ethanol (60%, 70%, 80%, 90%, 95% and 100%; Penta s.r.o., Prague, Czech Republic). After the drying process, the samples were treated and visualized as aforementioned. For the fluorescent microscopy, the fixed scaffolds were rinsed with PBS and incubated for 15 min with DAPI (1 mg of DAPI/1 mL of PBS, diluted 1:1000 in PBS prior to use, Sigma Aldrich) at room temperature in the dark. After the incubation period, the samples were rinsed with PBS and analyzed using the Nikon Eclipse Ti-E fluorescent microscope (Nikon Instruments Europe B.V., Amsterdam, Netherlands).

### In vivo study of the scaffold

#### Animal model and animal care

Sixteen New Zealand White rabbits (2.9 ± 0.7 Kg), 3.5 months old, were obtained from a conventional breed (Velaz spol. s.r.o., Unetice, Czech Republic) and bred in standard cages without bedding. The rabbits were fed ad libitum using the standard granular diet for rabbits (Altromin Spezialfutter GmbH & Co. KG, Lage, Germany). No protein or collagen supplements were administered to avoid confounding the effect of the scaffold on wound healing. The Ethical Principles and Guidelines for Scientific Experiments on Animals were respected throughout this study. The maintenance and handling of the experimental animals followed EU Council Directive 2010/63/EU, and the animals were treated in accordance with the principles of Care and Use of Animals. The investigation was approved by the Expert Committee of the Institute of Physiology, Academy of Sciences, Prague, Czech Republic, and conformed to Czech Animal Protection Law No. 246/92.

#### Surgical procedure and animal care

A total of 16 rabbits were randomly divided into two groups. The experimental group was treated with the microfibrous scaffold, the control group was left without any treatment. The animals were premedicated with intramuscular 15 mg diazepam *pro toto* (posterior thigh – semitendinous and semimembranous muscles). The surgical procedure was conducted under general anesthesia using ketamine (35 mg/kg) and xylazine (3 mg/kg) and subsequent inhalation of O_2_ + 1.5–2.0.5.0% Halothane during the surgery. All veterinary drugs were obtained from Veterinarni zasobovani spol. s.r.o., (Prague, Czech Republic).

**Fig. 2:**
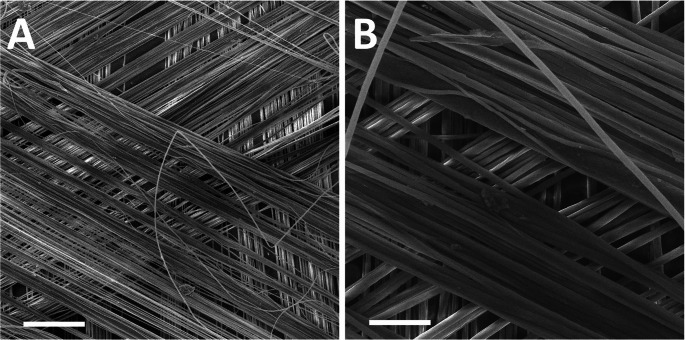
PCL fibers drawn in three dimensions visualized by scanning electron microscopy. Magnification 100✕, scale bar 500 μm (**A**), magnification 500✕, scale bar 100 μm (**B**)

After overlaying the surgery field with the sterile surgery cover, a skin incision in *linea alba* was made. The cut began above *processus xiphoideus* and continued in a caudal direction at a length of approximately 10 cm. The incision of the abdominal wall was also made in *linea alba*, and it began in a 2 cm caudal direction from *Cartilago xiphoidea* and continued in a caudal direction to a length of 5 cm. In the control group the tissue defect in the fascia was primarily closed using a 4/0 Monoplus suture (B. Braun Melsungen AG, Melsungen, Germany). In the experimental group the defect in the fascia was closed using a 4/0 Monoplus suture and the scaffold was placed over the fascia in an onlay position (Fig. 2A). The scaffold was then fixed with a continuous suture technique, using a 4/0 Monoplus suture and with single stitches in the reinforced points (Fig. 2B). In both groups the subcutis and subsequently the skin was closed using a 3/0 PDSII suture (Ethicon Inc, Somerville, NJ, USA). Antibiotics (cefalexinum monohydricum, 20 mg/kg/day subcutaneously) and analgesics (butorphanol tartrate, 0.1 mg/kg/day subcutaneously) were administered during the first 5 days. The rabbits were not limited in their movement after surgery. The animals were euthanized using T61 (Schering-Plough Corporation, Kenilworth, NJ, USA) 6 weeks later. The abdominal wall was explanted (Fig. 2C). Two strips of full layer abdominal wall, 2 cm wide and 6 cm long, were cut perpendicularly to the linea alba with an incision in the middle for the biomechanical tests. One sample from the suture line and one from the edge of the scaffold were harvested for histological testing. Samples for histological and immunohistochemical analysis were fixed in 10% phosphate-buffered formalin for 48 h. All residual suturing material was explanted prior to all tests.

#### Biomechanical testing of the regenerated rabbit abdominal wall

An elasticity in traction (E; N.mm^− 2^), the ultimate strength, which corresponds to the maximum stress that the specimen can withstand (σmax; N.mm^− 2^), and the corresponding proportional elongation value (εmax) were measured to characterize the biomechanical properties of the regenerated abdominal wall. The preparation of the sample and setup for biomechanical testing is described in detail in [15]. Briefly, the specimen (1 × 6 cm strip of abdominal wall) was fixed in grips of a MicroTester digital tension-meter, developed in the Department of Anatomy and Biomechanics, Faculty of Physical Education and Sport, Charles University in Prague (Utility model with document/registration number 25008, Industrial Property Office, Czech Republic). The samples were stretched by 5 mm at a speed of 10 mm/second ten times and were then pulled at a speed of 0.5 mm/second until the sample broke. The force response of each sample was detected at the branches of the tension-meter during the whole cycle, and the structure of the sample was monitored using a SZX-12 microscope (Olympus, Tokyo, Japan) equipped with an ultrasensitive camera (SensiCam; PCO, Kelheim, Germany).

#### Histological processing and quantification

*Histological processing.* The formalin-fixed tissue samples of the abdominal wall without skin were analyzed; two different samples from each animal: (i) abdominal wall without an incision, approximately 20 mm laterally to the median incision, and (ii) abdominal wall with an healing incision, either treated with simple suture and supported by the scaffold (experimental group; *n* = 8), or treated by the simple suture (control group; *n* = 8). Tissue samples were dehydrated and embedded in paraffin blocks. At least eight serial 5-µm-thick histological sections were processed from each paraffin-embedded tissue block. Two sections were stained with Verhoeff’s haematoxylin (Merck KGaA, Darmstadt, Germany) and green trichrome (DiaPath, Martinengo, Italy) to visualize the connective tissue [31]. Two sections were stained by picrosirius red (Direct Red 80, Sigma-Aldrich, St. Louis, MO, USA) diluted in saturated picric acid solution (Sigma-Aldrich, St. Louis, MO, USA) for one hour to visualize the collagen type I. The other two sections were processed immunohistochemically in order to reveal the presence of vascular, smooth muscle cells (SMC) in the microvessels (except capillaries) and myofibroblasts. We used α-smooth muscle actin as a marker of the contractile SMC phenotype and myofibroblasts. Endogenous peroxidase activity was blocked with 3% H_2_O_2_ in PBS. Unspecific binding activity was blocked with normal goat or horse serum in PBS for 20 min at room temperature. Following a pre-treatment for 20 min at 96 °C (Dako Target Retrieval Solution, pH 9; DakoCytomation, Glostrup, Denmark), the sections were incubated overnight at 4 °C with monoclonal mouse anti-human smooth muscle actin primary antibody (dilution 1:100, clone 1A4, DakoCytomation). Following the pre-treatment with enzyme-induced epitope retrieval with Proteinase K (DakoCytomation), another two sections were stained with monoclonal mouse anti-human and anti-rabbit CD31 antibody (clone JC/70A, dilution 1:40, Vector Laboratories Ltd., Burlingame, CA, USA). This staining was used to reveal the endothelium of the microvessels. Products of the immunoreaction were detected using the immunoperoxidase technique (N-Histofine kit; Nichirei Bioseciences, Tokyo, Japan) and the reactions were visualized with diaminobenzidine (Fluka, Buchs, Germany). All sections were counterstained with Gill’s haematoxylin.

*Histological quantification.* All quantitative estimates were done using well established stereological methods and Ellipse software (ViDiTo, Kosice, Slovakia). Sampling of histological sections and microscopic image fields, representing each tissue sample and used for estimating all the quantitative parameters, are summarized in Table 1. The sampling strategy relied on collecting multiple micrographs per each staining method from the same region of interest, as described in detail by Kolinko et al. [32].

**Table 1:** Sampling of histological sections and microscopic image fields for the Estimation of the quantitative parameters of each tissue sample

Quantitative parameter	Microscope objective used	Image fields sampled per section for quantification	Possible biological interpretation
*A*_*A*_*(collagen I*,* incision)*	10×	4	High fraction of mature type I collagen fibers most likely contributes to the mechanical resistance of the tissue.
*A*_*A*_*(collagen I*,* no incision)*	10×	4	
*A*_*A*_*(actin*,* incision)*	10×	2	High fraction of contractile myofibroblasts most likely contributes to the wound contraction.
*A*_*A*_*(actin*,* no incision)*	10×	2	
*Q*_*A*_ *(CD31 + microvessels*,* incision)*	10×	2	High density of microvessels most likely contributes to the regeneration and growth of the healing tissue.
*Q*_*A*_ *(CD31 + microvessels*,* no incision)*	10×	2	

Notes: The microscope objective and the magnification used for quantitative assessment of each of the parameters was the lowest one which permitted an exact and unambiguous identification of the counting events with respect to the histological staining methods. The reference space was the connective tissue below the dermis and above the abdominal muscle fascia

We used six continuous variables to describe the tissue reaction of the connective tissue below the dermis and superficial to the abdominal muscle fascia. The biological meaning of these variables is suggested in Table 1. The presence of type I collagen was assessed in the sections stained with picrosirius red using circularly polarized light. According to the thickness, type I collagen fibers are shown in yellow (thinner fibers), orange, and red color (thick bundles of fibers).

The presence of α-smooth muscle actin-positive cells was quantified in immunohistochemical sections. A randomly positioned uniform grid of equidistant points was placed on the micrographs in an overlay so the number of points hitting the type I collagen and the α-smooth muscle actin-positive cells were proportional to their area. We counted the number of points hitting these structures within the micrographs sampled from the area of the abdominal scar with the incision, as well as in the area without incision. The area of each major tissue component A was calculated by multiplying the number of counted points by the area corresponding to each point [6]. The presence of each tissue component under study was then expressed as their area fraction (A_A_) within the connective tissue of the scar and abdominal wall.

The presence of CD31-positive microvessel profiles was assessed in immunohistochemical sections using the projection of an unbiased counting frame consisting of two admittance and two forbidden borders [33], and we expressed the quantity of microvessels as the number of microvessel profiles per section area (Q_A_).

### Statistical analysis

The quantitative histological data were processed using Statistics Base 9 (StatSoft Inc, Tulsa, OK, USA). As a measure of the statistical relations between the variables, Spearman rank order correlations were used. Kruskal-Wallis ANOVA and the Mann Whitney U test were used for testing the equality of population medians between the groups under study. For paired samples with and without incision in the same animals, we used the Wilcoxon matched pairs test. Values were considered statistically significant for *p* < 0.05. Only significant findings and findings close to significant values are reported.

Quantitative data obtained from the in vitro tests and biomechanical assay are presented as mean ± standard deviation (SD). In MTT assay the average values were determined from 4 independently prepared samples. The results were evaluated statistically using One Way Analysis of Variance (ANOVA) and the Student-Newman-Keuls Method. The level of significance was set at 0.001 and 0.05.

## Results

### Characterization of the drawn fibers

**Fig. 3:**
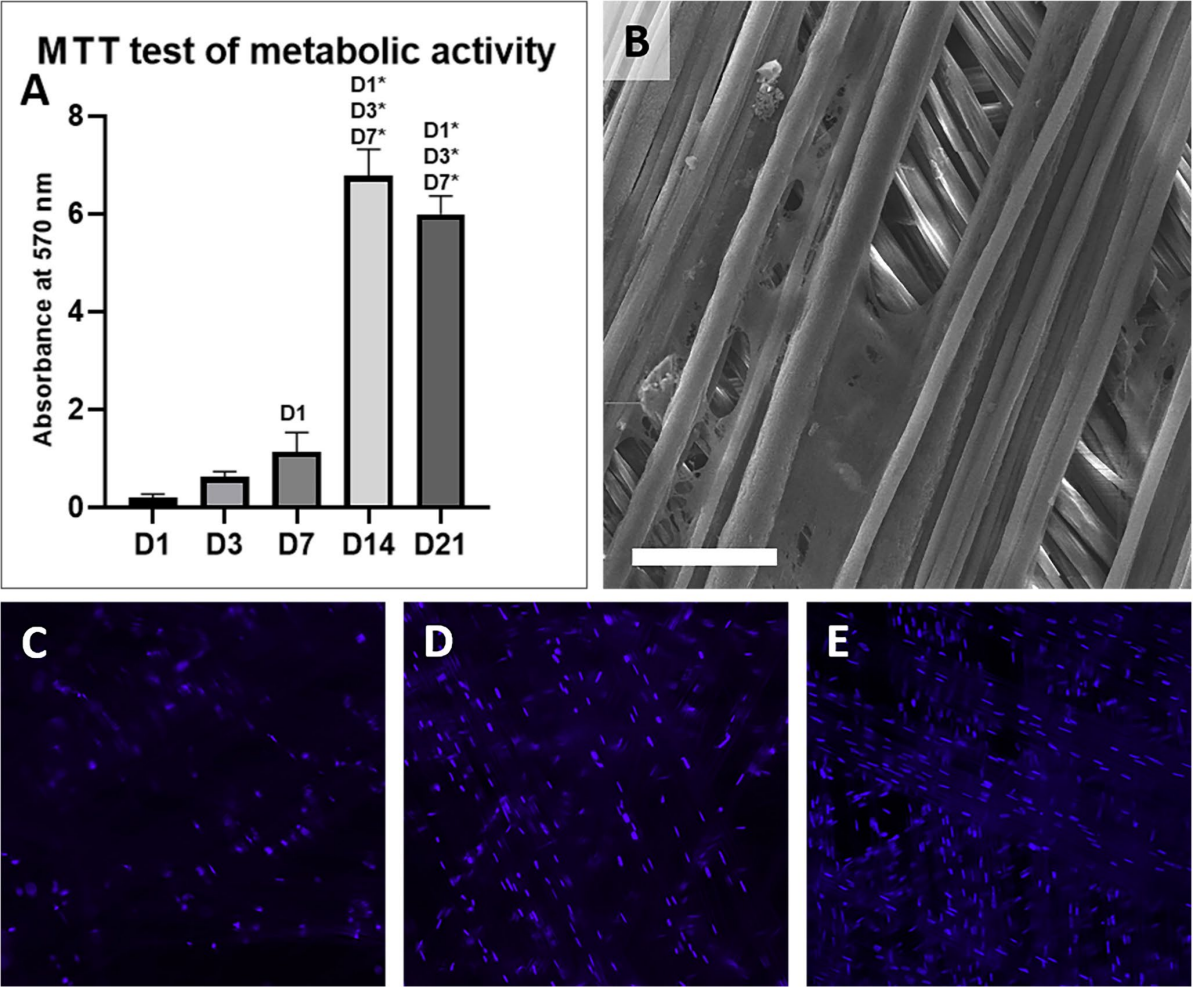
In vitro test of drawn fiber biocompatibility. The cell metabolic activity of 3T3 fibroblasts cultured on the PCL microfibers increased during the experiment (**A**) (n=4; p<0.05, p<0.001 is indicated by *). The 3T3 fibroblasts adhered on the fibers were visualised using SEM on day 21 (**B**; magnification 500✕, scale bar 100 μm). The cell distribution was detected using DAPI staining and visualization using fluorescent microscopy (**C**, **D**, **E**; magnification 10✕, scale bar 100 µm). It is visible that the cell number increased on day 7 (**D**) and 21 (**E**) compared to day 1 (**C**)

The fibers prepared using the drawing technique were visualized using SEM (Fig. 3). The fibers were smooth and oriented parallel to each other in three dimensions. Stereological analyses showed that the PCL fibers had an average diameter 8.70 ± 5.33 μm.

### In vitro test of PCL fiber biocompatibility

**Fig. 4:**
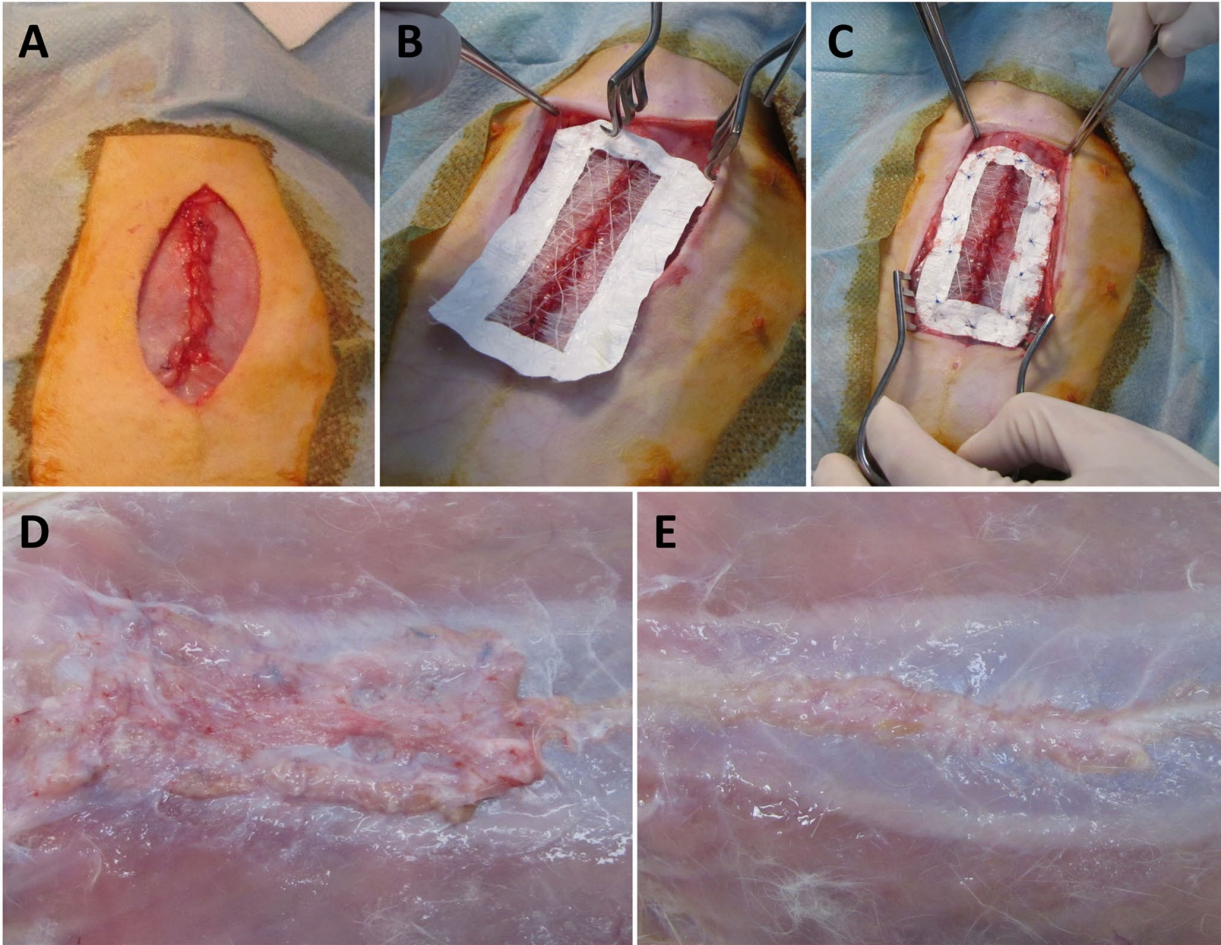
In vivo test of the composite scaffold. The incision in linea alba was sutured (**A**) and left without any further treatment in the control group. Alternatively, the scaffold was applied on the incision (**B**) and fixed to the abdominal wall (**C**). The abdominal wall with implanted scaffold (**D**) or without (**E**) was explanted after 6 weeks and examined from a biomechanical and histological perspective

The cell metabolic activity was evaluated by the MTT assay on days 1, 3, 7, 14 and 21 (Fig. 4A). The measured absorbance gradually substantially increased until day 14 and then stagnated. This reflects the fact that the cells had already formed a confluent layer on the fibers and contact inhibition occurred. The visualization using SEM showed good cell adhesion; cells adhered on the fibers and spread along them (Fig. 4B). Fluorescence microscopy visualization showed the nuclei of the adhered cells with evidently increasing numbers during the 21 days of the experiment (Fig. 4C, D and E). This corresponds with the data from the MTT test and confirmed the biocompatibility of the drawn fibers.

### In vivo study

The drawn microfibers were combined with a nanofibrous frame to create a scaffold for the incisions. The scaffold was implanted in the abdominal incision of rabbits. All the animals survived until euthanasia, which was performed 6 weeks after implantation without any visible changes of their condition. The regenerated abdominal walls did not show any sign of pathological changes or inflammation. All the incisions were healed without formation of incisional hernia.

#### Biomechanical evaluation

The examination of the biomechanical properties of the regenerated abdominal wall showed a higher elasticity of tension in the group treated with scaffold compared to the control group.

The 1 × 6 cm strips of the regenerated abdominal wall of each animal, attached by the grips of the tension meter to the healthy abdominal muscles, were biomechanically tested, as described in the Methods section. The force response and hysteresis curves of the explanted abdominal walls were analyzed. Average values of the biomechanical quantities are summarized in Table 2.

**Table 2:** Average values of the Biomechanical quantities (*n* = 8)

Group	E [*N*/mm^2^]	σ_max_ [*N*/mm^2^]	ε_max_ [-]
Non-treated incision	1.44 ± 0.33	0.66 ± 0.33	0.79 ± 0.2
Incision treated with scaffold	2.54 ± 0.84	0.86 ± 0.21	0.73 ± 0.14

**Fig. 5:**
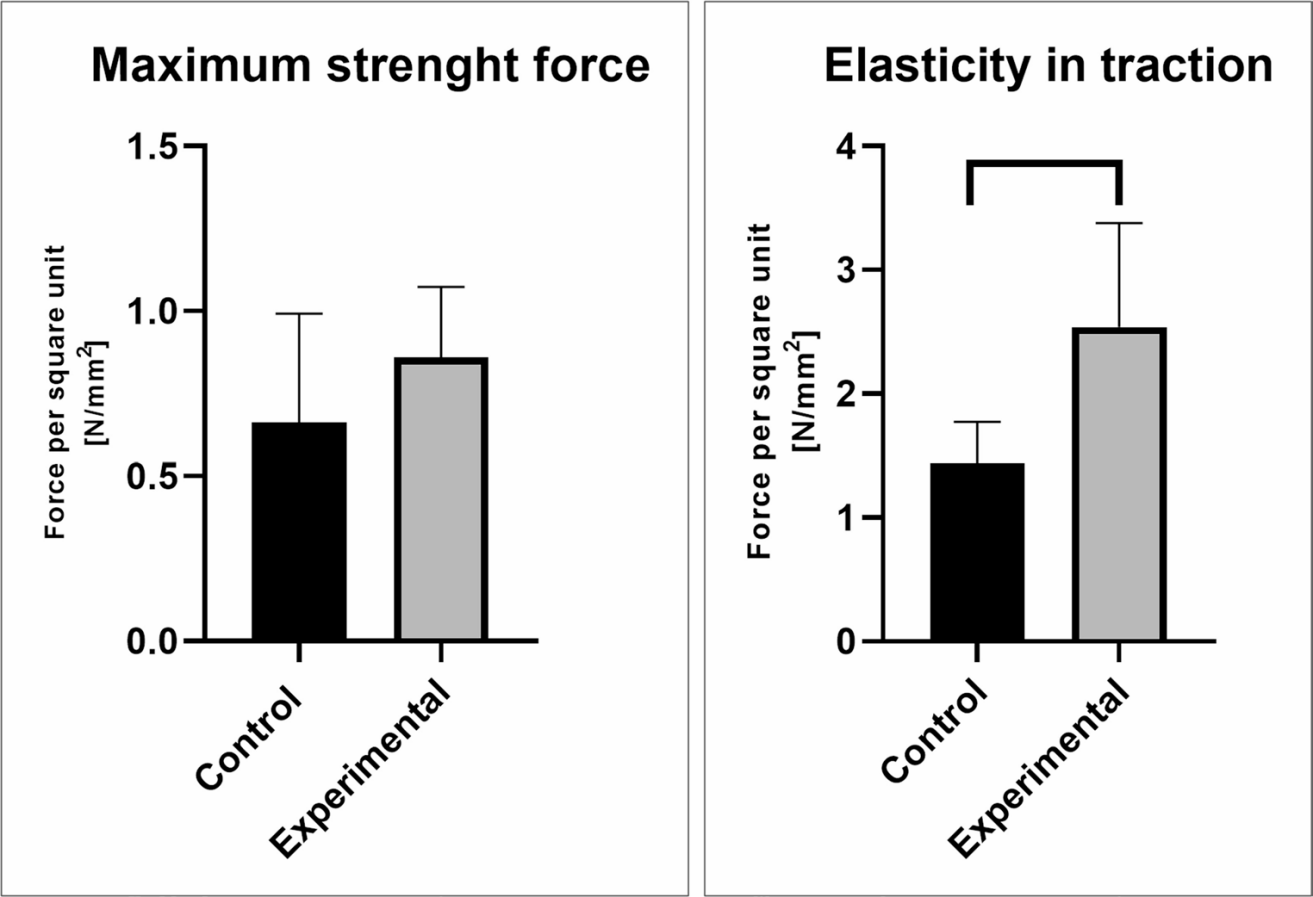
Average values of the elasticity in tension and maximal stress of the non-treated incision and the incision treated with scaffold 6 weeks after surgery. The maximal stress of the tore sample was on the same level but the elasticity in traction of the incision treated with the scaffold reached a significantly higher value. The level of statistical significance for the assays is designated above the mean values (*n* = 8; *p* < 0.05)

We compared the average values of the elasticity in tension and maximal stress of the non-treated incision and the incision treated with microfibrous scaffold 6 weeks after surgery (Fig. 5). The maximal stress of all tore samples did not achieve significant changes among the average values. However, the biomechanical analysis revealed significantly higher values of elasticity in the tension of the samples where the incision was treated with the scaffold.

#### Histological evaluation

Two tissue blocks from each animal were evaluated. One sample was collected from the healing incision site, which was either treated with a simple suture or with a microfiber scaffold; the second sample was taken from the surrounding area, which was either covered or not covered with the scaffold. The surrounding area was defined as the region approximately 20 mm lateral to the midline incision. Each sample contained several layers, including subcutaneous fat, abdominal muscles and their fasciae, extraperitoneal fat, and the parietal peritoneum. Although some regions of the abdominal fasciae were fused, the layers of abdominal skeletal muscles generally retained their organization, with only small and sporadic muscle bundles displaced at the healing incision site.

In samples containing scaffolds, remnants of the scaffold were typically better preserved in areas without an incision, whereas they appeared more resorbed within the incision site. The scaffold remnants themselves contained only small amounts of collagen but were usually surrounded by abundant collagenous connective tissue. Average histological values are summarized in Table 3, and all experimental data are provided in Supplement 1.

**Table 3:** Average values of the histological parameters

Quantitative parameter	Scaffold	No scaffold	*p*-value
*A*_*A*_*(collagen I*,* incision)*	0.14 ± 0.5	0.07 ± 0.03	0.007
*A*_*A*_*(collagen I*,* no incision)*	0.04 ± 0.04	0.03 ± 0.02	-
*A*_*A*_*(actin*,* incision)*	0.54 ± 0.85	0.14 ± 0.08	-
*A*_*A*_*(actin*,* no incision)*	0.24 ± 0.15	0.04 ± 0.03	0.014
*Q*_*A*_ *(CD31 + microvessels*,* incision) (mm*^*− 2*^*)*	12.5 ± 4.04	12.32 ± 5.49	-
*Q*_*A*_ *(CD31 + microvessels*,* no incision) (mm*^*− 2*^*)*	8.49 ± 3.68	4.51 ± 0.84	-

**Fig. 6:**
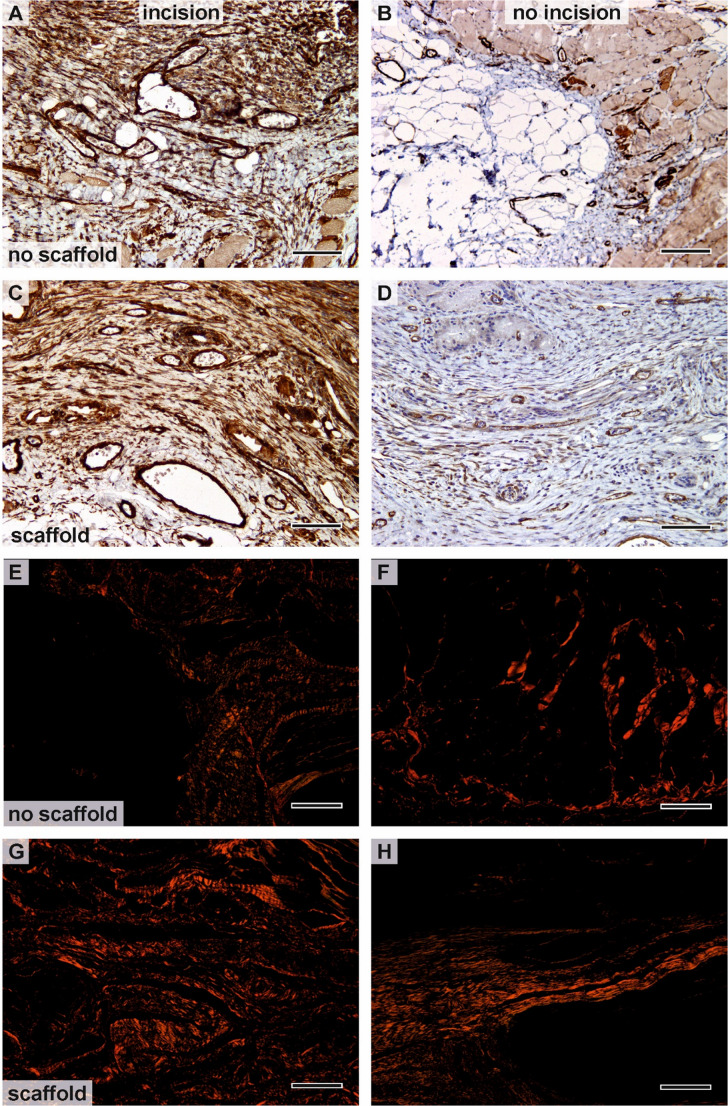
The α-smooth muscle actin immunohistochemical evaluation of the samples and visualization of the type I collagen stained with the picrosirius red in the polarised light. (**A**, **E**) Incision in the tissue samples uncovered with the scaffold. (**B**, **F**) The surrounding area uncovered with the scaffold. (**C**, **G**) Incision in the tissue samples covered with the scaffold. (**D**, **H**) The surrounding area covered with the scaffold. The incision covered with the scaffold (**C**) contained a comparable amount of myofibroblasts to the samples with no covering (**A**). The surrounding area without scaffold covering contained very few myofibroblasts (**B**). Wounds covered with the scaffold showed a more regular distribution of type I collagen (**G**, **H**) compared to uncovered defects (**E**, **F**). (**A**, **B**, **C**, **D**) scale bar 100 μm; (**E**, **F**, **G**, **H**) scale bar 200 μm

Type I collagen was stained with picrosirius red and visualized under polarized light (Fig. 6). In samples where either the incision or the surrounding area was not covered with the scaffold, variable amounts of type I collagen were observed, often interrupted by gaps corresponding to adipose tissue (Fig. 6E, F). The visualized collagen appeared in shades ranging from red to yellow. Histological evaluation of the scaffold-supported tissue revealed a more uniform distribution of type I collagen and a higher collagen fraction (Fig. 6G, H) compared to samples without scaffolds (Fig. 6E, F).

Immunohistochemical staining for α-smooth muscle actin–positive cells identified myofibroblasts within the healing scar and vascular smooth muscle cells in the microvessels. All samples—particularly those in which either the incision or the surrounding area was covered with the scaffold (Fig. 6C, D) and the uncovered incision (Fig. 6A)—contained variable numbers of myofibroblasts (dark brown staining) and blood vessels within the granulation tissue. The numbers of myofibroblasts were comparable among these samples. In contrast, the unhealed tissue, i.e., the area not covered by the scaffold, consisted mainly of truncal fascia and adipose tissue overlying the abdominal muscles, with only a few myofibroblasts present (Fig. 6B).

**Fig. 7:**
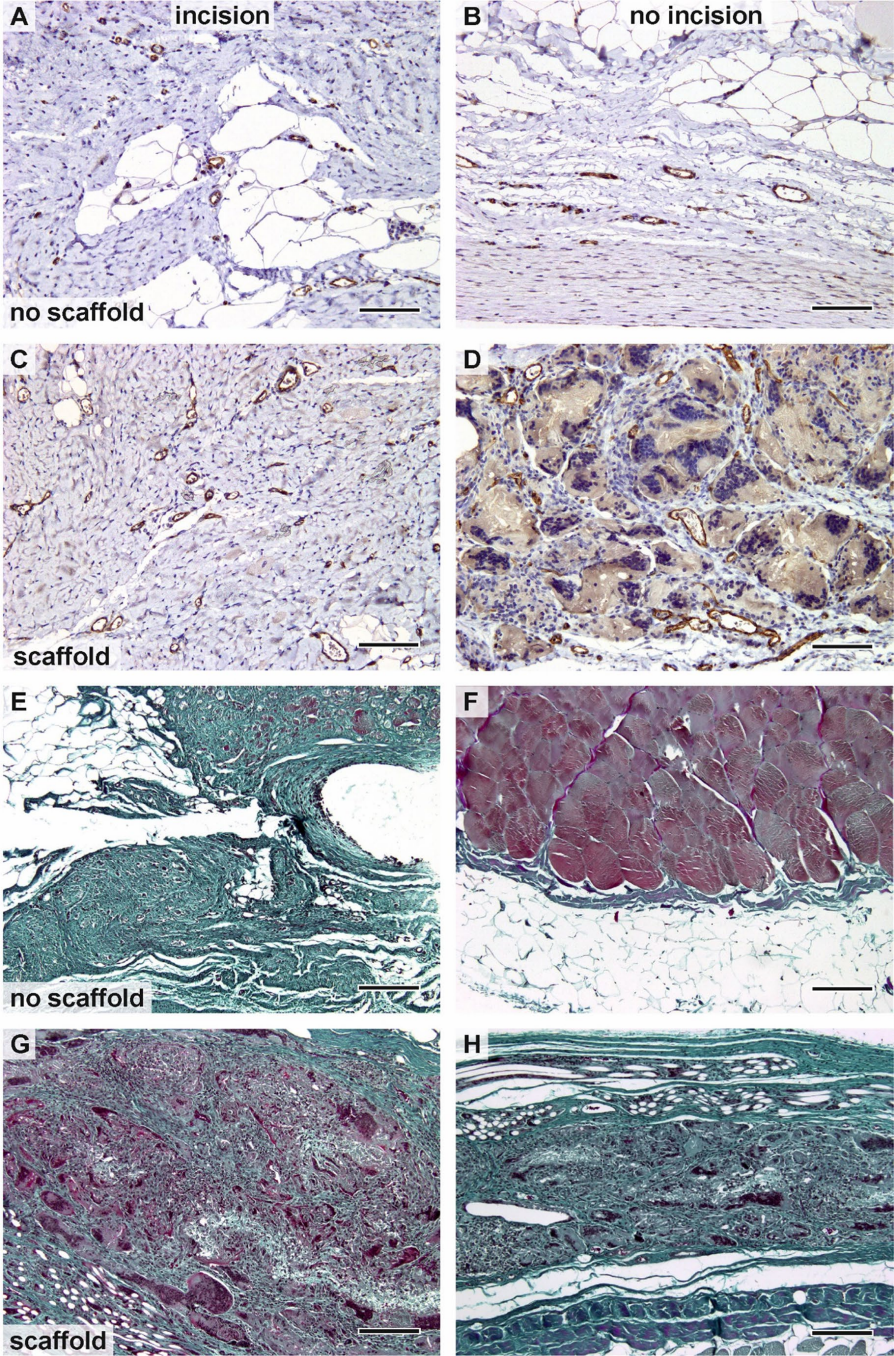
Evaluation of microvessels within the samples using CD31 immunohistochemistry and overall trichrome staining of the samples. (**A**, **E**) Incision in the tissue samples uncovered with the scaffold. (**B**, **F**) The surrounding area uncovered with the scaffold. (**C**, **G**) Incision in the tissue samples covered with the scaffold. (**D**, **H**) The surrounding area covered with the scaffold. The density of microvessels in the incision was comparable in the samples with (**C**) and without scaffold (**A**). The surrounding area covered with scaffold (**D**) contained more microvessels than the samples without covering (**B**). The tissue of the incision was less organized and contained adipose tissue (**E**, **F**). Wounds supported with scaffolds showed good integration of the scaffold both with granular connective tissue and dense collagenous connective tissue (**G**, **H**). Scale bar 100 μm

Endothelial cells in the microvessels were visualized using CD31 immunohistochemical staining (Fig. 7). The mean number of microvessels within the incision site was comparable between samples with (Fig. 7C) and without scaffolds (Fig. 7A). In the surrounding area, a higher density of microvessels was observed in samples covered with the scaffold (Fig. 7D), where the vessels were often associated with scaffold remnants, compared with the uncovered surrounding area (Fig. 7B).

Green trichrome and Verhoeff’s hematoxylin staining demonstrated the overall tissue organization (Fig. 7). The tissue around the incision that was not supported by the scaffold appeared less organized and contained variable amounts of adipose tissue (Fig. 7E). The uncovered surrounding area consisted mainly of truncal fascia and adipose tissue overlying the abdominal muscles (Fig. 7F). In contrast, the incision covered with the scaffold showed good integration of the scaffold with both granulation tissue containing leukocytes and dense collagenous connective tissue (Fig. 7G). Remnants of the scaffold were also present in the regions without incision (Fig. 7F, H).

**Fig. 8:**
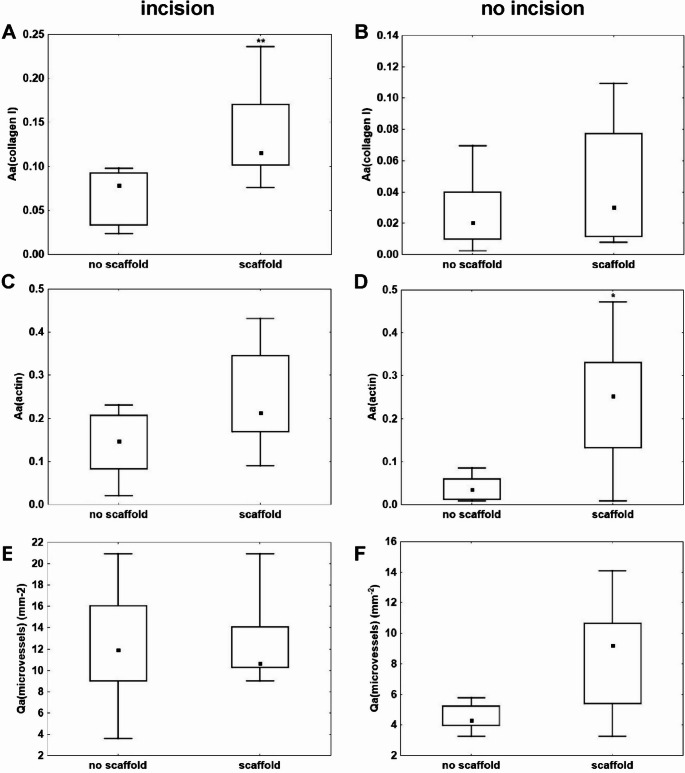
Paired comparison of the samples covered and uncovered with scaffold. Left column - differences between the healing incision treated with scaffold or without the treatment. The incision treated with scaffold contained a greater fraction of type I collagen (**A**). (**A**) Fraction of type I collagen in the healing incision. (**C**) Fraction of actine-positive myofibroblasts (actin) in the healing incision. (**E**) Density of microvessels in the healing incision. Right column - differences between the healing surrounding areas covered with scaffold or without covering. The tissue of the surrounding area covered with scaffold had a greater fraction of myofibroblasts (**B**). (**B**) Fraction of type I collagen in the healing surrounding area. (**D**) Fraction of myofibroblasts (actin) in the healing surrounding area. (F) Fraction of microvessels in the healing surrounding area. (*n* = 8; *p < 0.05)

The histological sections from control and experimental animals and different areas of the abdominal wall were compared in this quantitative study. The quantitative histological evaluation between the healing incisions treated either with scaffold or the simple suture revealed a greater fraction of type I collagen in animals with scaffold treated incisions (p˂0.01; Fig. 8A).

The healing abdominal wall was either covered with the scaffold or was kept without any treatment. The tissue covered with the scaffold contained a greater fraction of myofibroblasts in comparison with the uncovered surrounding area (p˂0.05; Fig. 8D). We did not observe any significant changes in the other histological parameters, such as the fraction of myofibroblasts in the incision tissue (Fig. 8C) and the content of type I collagen in the surrounding area (Fig. 8B) when we compared the samples covered or uncovered with the scaffolds. In addition, neither compared group of samples contained significantly different densities of microvessels (Fig. 8E, F).

**Fig. 9:**
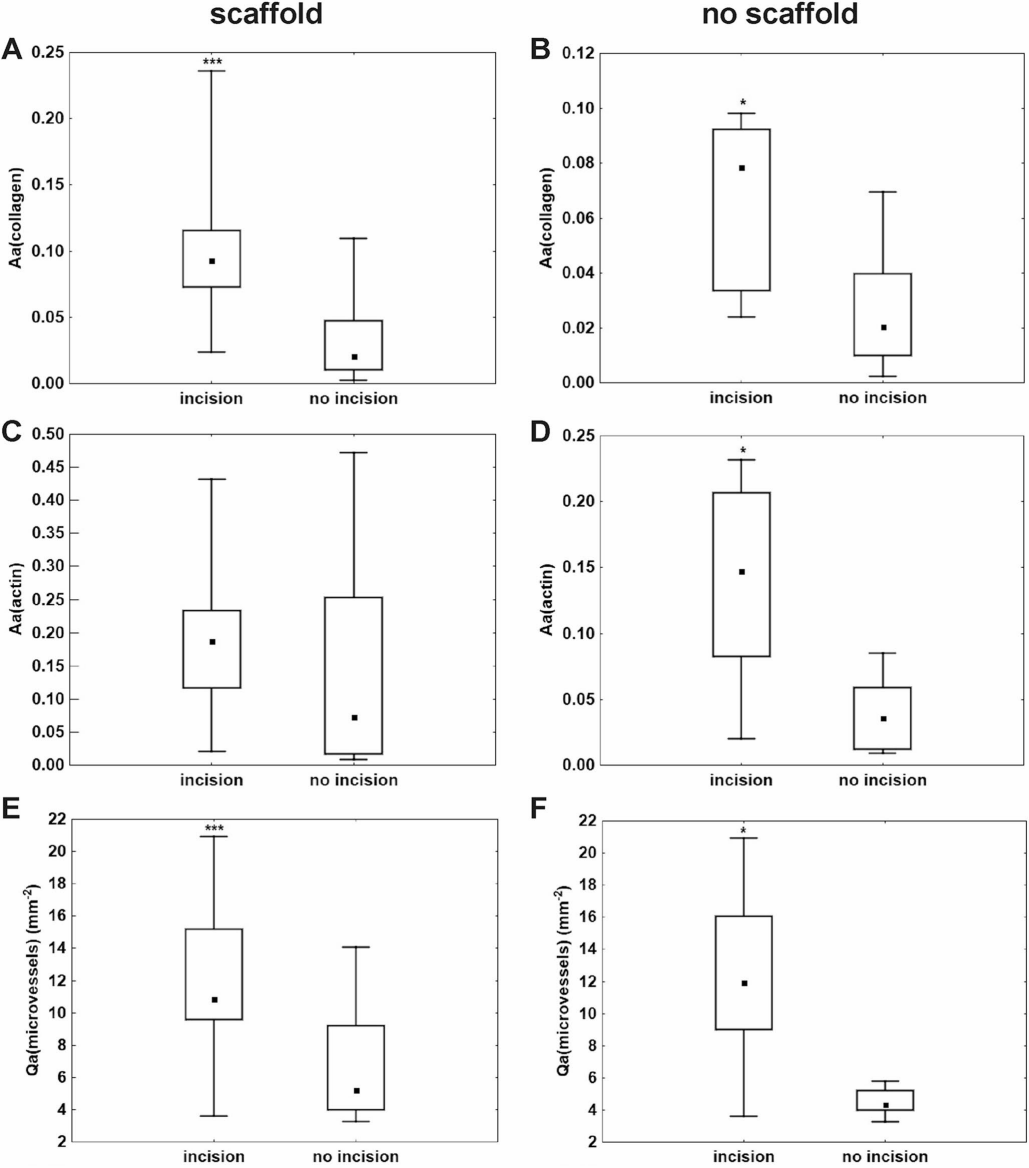
Paired comparison of the healing incision and the surrounding area. Left Column - all the samples were covered with the scaffold. The incisions contained greater fraction of type I collagen (**A**) and higher density of microvessels (**E**). (**A**) The quantitative histological evaluation of the content of type I collagen. (**C**) The quantitative histological evaluation of the content of myofibroblasts (actin). (**E**) The quantitative histological evaluation of the microvessel density. Right column - all the samples were uncovered with the scaffold. The incisions contained greater fraction of type I collagen (**B**), greater fraction of myofibroblasts (**D**) and a higher density of microvessels (**F**). (**A,B**) The quantitative histological evaluation of the content of type I collagen. (**C,D**) The quantitative histological evaluation of the fraction of myofibroblasts. (**E,F**) The quantitative histological evaluation of the microvessel density. (*n* = 8; *p < 0.05)

A paired comparison of the quantitative histological evaluations is presented in Fig. 9. The tissues from the healing incision and the surrounding area were compared in samples either covered with the scaffold (Fig. 9A, C, E) or left uncovered (Fig. 9B, D, F).

In the scaffold-covered incisions, both the fraction of type I collagen (*p* < 0.001; Fig. 9A) and the density of microvessels (*p* < 0.001; Fig. 9E) were significantly higher compared with the scaffold-covered surrounding area.

In the uncovered samples, significantly higher values were observed for all evaluated parameters—specifically, the fraction of type I collagen (*p* < 0.05; Fig. 9B), the fraction of myofibroblasts (*p* < 0.05; Fig. 9D), and the density of microvessels (*p* < 0.05; Fig. 9F)—in comparison with the corresponding surrounding tissue. Nevertheless, the *p*-values for the paired comparison between the covered incision and surrounding area were below 0.001, whereas for the uncovered samples they were only below 0.05.

## Discussion

### Advantages and technical specifics of the fiber-drawing method

Drawing is a unique method for the preparation of microfibers, with precise orientation. The orientation of the fibers promotes coordinated cell growth in a particular direction. Drawing has been described and used previously [20, 30], but it has not attracted much attention of late. One of the reasons for this could be the low productivity of the fibers compared to other nonwoven technologies, e.g. electrospinning, forcespinning, melt-blown and others, which are often used for the fabrication of micro- or nanofibers from various types of polymers [24]. They are reasonably productive, nevertheless they can produce oriented structures only in layers, and often the orderliness is not so accurate. Recently, drawing was used to fabricate various random and oriented polymer structures by Tokarev et al. [34], and by Yuan et al. [23]. Tokarev et al. call their method a brush spinning technique, since he was first using a rotating hairbrush as the drawing device (the bristles of the brush touched the polymer droplet and elongated the fiber) as well as the collector of fibers. Later, he used collectors of defined shapes (tubes, cubes). Even though this approach provides highly oriented structures, the fibers are prepared in layers and the single fibers cannot be manipulated. Yuan et al. drew the fibres one by one in specific directions on the underlay, to build fibrous support to grow blood capillaries. His approach is very similar to our drawing method, which enables us to manipulate with a single fiber while drawing.

The aim of our study was to develop a scaffold with a precise fiber orientation perpendicular to the abdominal incision. The fibers would allow the growth of cells along them, and the formation of an extracellular matrix that would promote and accelerate scar healing. For the primary in vitro test, fibers were attached to an insert and crossed in three directions to increase cell seeding efficiency. The drawing process enabled the production of smooth fibers with a diameter in a range of microfibers (8.70 ± 5.33 μm).

### Biocompatibility and cellular response to drawn microfibers

The in vitro test showed good biocompatibility of the fibers. Both the MTT assay and the fluorescence microscopy show very good cell attachment on day 1 of the experiment. The MTT assay shows exponential cell growth with a peak on day 14. From day 21 the metabolic activity stagnated, which could be because the cells had already covered the whole sample and stopped proliferating as a result of contact inhibition. This is in accordance with the fluorescence images.

PCL, in the form of nano- or microfibers, is widely used in tissue engineering applications as it is a biocompatible and biodegradable polymer and is approved for medical use. PCL nanofibers have been shown in many studies to be a perfect scaffold for cell growth, with electrospinning being the most commonly used method of preparation. Among other methods, melt blowing or centrifugal spinning can be used to prepare the fibrous structured scaffolds [35–37]. Chen et al. demonstrated the biocompatibility of electrospun PCL nanofibers for 3T3 fibroblasts and showed that cell adhesion and proliferation are dependent on fiber diameter [38]. Other cell types, such as mesenchymal stem cells or keratinocytes, have also been successfully cultured on PCL nanofibers [39].

The oriented morphology of fibrous scaffolds gives them the ability to guide cell migration. This phenomenon makes it possible to direct cell growth in the desired direction. This can be used as an advantage in some tissues, such as vascular endothelial or neural tissue and also in wound healing applications [40, 41]. Aligned fibers can be produced by electrospinning or force spinning using special collectors [37]. However, drawing technology allows more precise placement of the fibers.

### In vivo performance: tissue regeneration and mechanical strength

The scaffolds for the in vivo study had to be designed so that the parallel fibers overlapped the scar. To accomplish this, drawn fibers were attached to frames prepared from PCL electrospun nanofibers. Such a composite scaffold allowed good manipulation during implantation. The scaffold was fixed to the abdominal wall with several sutures to prevent its movement. The scaffolding design proved successful. The scaffold was fixed to the implant site throughout the experiment. We did not observe any signs of inflammation or immune response, indicating good biocompatibility of the scaffold.

Histological examination showed that incisions supported by the scaffold developed a more typically organized wound structure, with a higher content of type I collagen than spontaneously healing incisions without scaffold support. The scaffolds were well integrated into both the granulation and dense connective tissue. The inflammatory cells adjacent to the scaffolds did not form foci that might weaken the abdominal wall.

The scaffolds also appeared to influence blood vessel formation in the adjacent, unincised region, resulting in a greater proportion of myofibroblasts in rabbits with scaffolds compared to those without. In both treatment groups—whether the incision was supported by a scaffold or not—the incision area exhibited a higher percentage of type I collagen and a significantly greater microvessel density than the surrounding areas, reflecting the normal healing response of injured tissue.

In scaffold-supported incisions, remnants of the scaffold were typically more resorbed within the incised area, with minimal preservation compared to the unincised region. This observation aligns with the scaffold design, in which the incision area was only partially covered by drawn fibers, in contrast to the dense nanofiber mesh of the electrospun scaffold. The specimen design thus proved advantageous: only fine fibers were present at the incision site to bridge the wound, while mechanical strength was provided by the nanofibrous frame with a higher density and a correspondingly slower degradation rate, which did not adversely affect the healing process.

Histological examination revealed increased microvessel formation in the region adjacent to the scaffold-treated incision. Angiogenesis, the process by which new blood vessels develop from pre-existing ones, contributes to the establishment of a functional vascular network within the wound site. Microvessel formation is essential for wound healing, as it supplies oxygen and nutrients to regenerating tissue, facilitates the transport of immune cells to the wound, and aids in the removal of inflammatory mediators, thereby promoting tissue repair [42].

Effective tissue regeneration relies on angiogenesis, which supports the formation of new tissue structures and contributes to the remodeling phase of wound healing. In our study, the area fraction of actin positively correlated with microvascular density in both the incision and the surrounding regions of scaffold-treated and untreated wounds. This finding supports the hypothesis that pericytes differentiate into myofibroblasts during the healing process [43].

The area fraction of type I collagen within the incision was higher in samples covered with the scaffold than in those without scaffold support, and the area fraction of actin in the surrounding tissue was also greater in scaffold-covered samples compared to uncovered ones. Collagen network stability is regulated by myofibroblast contractions: the tightening of previously secreted collagen fibers enables adjacent myofibroblasts to deposit additional collagen, thereby increasing the strength of the wound granulation tissue [44].

Type I collagen plays a critical role in all phases of abdominal wall wound healing by providing essential structural support during tissue repair. As the most abundant collagen in the body, it is crucial for the formation of a strong and well-organized extracellular matrix [45]. During the healing process, type I collagen fibers are deposited within the abdominal wall to restore tissue integrity and mechanical strength. Proper synthesis and remodeling of type I collagen are essential for long-term repair and for preventing complications such as hernia formation or wound dehiscence.

The orientation of the fibers perpendicular to the incision line was intentionally designed to guide cellular migration and extracellular matrix deposition along a mechanically relevant axis. Aligned fibrous scaffolds are known to promote directional fibroblast elongation and collagen fiber alignment, which can enhance the tensile properties of the healing tissue compared to random architectures [41, 46]. In our study, this concept was supported by histological findings showing denser and more organized collagen deposition in scaffold-treated wounds. Although the study was not designed to quantitatively distinguish the behavior of individual cell populations, the increased microvessel density and collagen fraction in scaffold-supported wounds suggest that both fibroblasts and endothelial cells infiltrated the scaffold effectively.

A high proportion of mature type I collagen fibers likely contribute to the increased mechanical strength of the repaired tissue. The fibrous scaffold further enhanced the mechanical properties of the scar six weeks after implantation. Mechanical testing revealed a significantly higher Young’s modulus in scaffold-treated incisions compared with untreated ones. This parameter reflects the tissue’s elasticity under tension and indicates its resistance to deform when subjected to tensile or compressive forces.

Although incisions treated with the scaffold showed significantly higher elasticity modulus, no clear increase in tensile strength was observed. This result suggests that in the early stages of healing, collagen fibers are better organized—they are more regularly oriented and interconnected, which increases tissue stiffness under physiological stress. This phenomenon is consistent with the histological evaluation, which showed a more regular arrangement of type I collagen and its greater representation in scaffolded samples compared to control samples.

However, when testing the mechanical properties using a tensile test, the sample may break at its weakest point. This means that the measured strength limit corresponds primarily to the properties of this specific zone. In our case, the area of lowest strength was mainly muscle tissue. It can therefore be assumed that the suture with the applied scaffold achieved higher local strength than the surrounding native tissue at the time of the experimental measurement, but this fact was not reflected in the overall tensile strength value.

From a biomechanical point of view, the scaffold improves the local organization of collagen and thus the functional stability of the scar within the elastic range of the load, but does not affect the overall strength limit of the entire muscle-fascial complex. Clinically, this effect can be interpreted as beneficial for the early phase of healing, when the scaffold supports the structured regeneration and may reduce the risk of dehiscence or subsequent hernia formation.

In addition to tensile strength and collagen remodeling, post-surgical adhesions (PSAs) represent another critical factor influencing the long-term outcomes of abdominal wall repair. PSAs can develop rapidly, with tenacious adhesions forming within one hour of injury, highlighting the importance of early preventive strategies during or immediately after surgery [47]. Myofibroblasts play a central role in adhesion formation by driving extracellular matrix deposition and peritoneal fibrosis [48]. Although the current study did not assess adhesion development, future research should include systematic macroscopic and histological evaluation of PSAs. Quantitative adhesion scoring, combined with imaging and molecular markers of fibrosis and angiogenesis, would provide a more comprehensive understanding of how the scaffold influences peritoneal healing. Such analyses could help clarify whether scaffold-assisted repair modifies the extent of adhesion, tissue organization, or the persistence of myofibroblasts in the long term.

### Limitations of the study

The relatively small number of animals per group (*n* = 8) reflects the current emphasis on the ethical reduction of experimental animal use in biomedical research. Despite this limited cohort, we observed statistically significant differences between the experimental and control groups, which underlines the robustness and reproducibility of the findings. A larger sample size might further accentuate these differences; however, such an increase would not be ethically justified given the clear statistical outcomes already obtained. The healing process was assessed at a single postoperative time point, which provides only a partial view of the temporal dynamics of tissue regeneration. The rabbit model remains widely accepted for studies of abdominal wall repair, although anatomical, biomechanical, and physiological differences, such as a thinner abdominal wall, lower intra-abdominal pressure, and distinct muscular architecture, create a less demanding mechanical environment compared to humans. Their uniform health status and genetic background also differ from the heterogeneity of human patients, who frequently present with comorbidities such as diabetes or obesity. Nevertheless, within these experimental constraints, the present study provides meaningful insight into the potential of fiber-drawn microfibrous scaffolds to enhance abdominal wall incision healing.

This work represents a preliminary study performed under laboratory-scale scaffold production conditions. We acknowledge that further optimization of scaffold fabrication, upscaling, and comprehensive preclinical evaluation—covering multiple time points, larger animal models, and biocompatibility testing under regulatory standards—will be required before any potential translation toward clinical application. Nevertheless, within these experimental constraints, the present study provides meaningful insight into the potential of fiber-drawn microfibrous scaffolds to enhance abdominal wall incision healing.

## Conclusion

In conclusion, our study demonstrates that the fiber-drawn microfibrous scaffold effectively supported incision healing by promoting collagen deposition and improving the mechanical properties of the regenerated abdominal wall tissue. Although no significant increase in ultimate tensile strength was detected, the observed enhancement of elasticity and the more organized collagen architecture indicate that the scaffold contributes to improved early structural integrity of the scar. From a biomechanical standpoint, the overall strength of the abdominal wall remains primarily determined by the properties of native muscle tissue, while the scaffold provides additional mechanical coordination and stability during the early stages of healing.

Given these findings, the scaffold design may be particularly beneficial for patients with impaired wound healing, such as those with obesity, diabetes mellitus, chronic systemic diseases, or long-term corticosteroid or immunosuppressive therapy. In such risk groups, early mechanical reinforcement and guidance of tissue regeneration could help prevent incisional hernia development and improve postoperative outcomes.

## Supplementary Information

Below is the link to the electronic supplementary material.

Supplementary Material 1 (DOCX 20.3 KB)
