# Supplementary material for: Enhancing abdominal wall healing using an oriented polycaprolactone microfibrous scaffold prepared using the fiber drawing method: A rabbit model study
Source: Hernia. 2026 Jan 16;30(1):67. doi: 10.1007/s10029-025-03544-z (PMC12811376; doi:10.1007/s10029-025-03544-z)
Supplement: Supplementary file 1 — Supplementary Material 1 (DOCX 20.3 KB) [file 10029_2025_3544_MOESM1_ESM.docx]

Supplementary Table 1: Experimental data of histological parameters
